# Controlling inter-particle distances in crowds of motile, cognitive, active particles

**DOI:** 10.1038/s41598-024-59022-6

**Published:** 2024-04-24

**Authors:** Rajendra Singh Negi, Priyanka Iyer, Gerhard Gompper

**Affiliations:** Theoretical Physics of Living Matter, Institute of Biological Information Processing and Institute of Advanced Simulation, Forschungszentrum Jülich, 52425 Jülich, Germany

**Keywords:** Biological physics, Statistical physics, thermodynamics and nonlinear dynamics, Computer modelling

## Abstract

Distance control in many-particle systems is a fundamental problem in nature. This becomes particularly relevant in systems of active agents, which can sense their environment and react by adjusting their direction of motion. We employ agent-based simulations to investigate the complex interplay between agent activity, characterized by Péclet number $\hbox{Pe}$, reorientation maneuverability $\Omega$, vision angle $\theta$ and vision range $R_0$, and agent density, which determines agent distancing and dynamics. We focus on semi-dense crowds, where the vision range is much larger than the particle size. The minimal distance to the nearest neighbors, exposure time, and persistence of orientation direction are analyzed to characterize the behavior. With increasing particle speed at fixed maneuverability, particles approach each other more closely, and exhibit shorter exposure times. The temporal persistence of motion decreases with increasing $\hbox{Pe}$, reflecting the impact of activity and maneuverability on direction changes. For a vision angle $\theta =\pi /4$, we observe the emergence of flocking aggregates with a band-like structure, somewhat reminiscent of the bands in the Vicsek model. Additionally, for vision angles $\theta \ge \pi /2$, several quantities are found to display a universal scaling behavior with scaling variable $\hbox{Pe}^{3/2}/\Omega$. Our results are in good agreement with recent experiments of pedestrians in confined spaces.

## Introduction

Controlling and keeping distances is a ubiquitous issue, both in condensed matter and in living systems. In liquid or crystalline condensed phases at thermal equilibrium, the distance between neighboring atoms or molecules is determined by the competition of short-range repulsive and longer-range attractive interactions^1^. In colloidal systems, interactions can be designed in many ways, and systems with unusual interactions, like short-range attractive and long-range repulsive, have been constructed^2^. The well-controlled condensed phases are important for many bulk material properties, like compressibility, shear modulus, electrical conductivity, etc. Interestingly, also purely repulsive interactions can lead to crystallization, such as in a gas of electrons moving in a uniform, inert, neutralizing background, where optimal distancing, determined by a minimum of the electrostatic energy, is found to be attained by the formation of a lattice structure—the Wigner crystal—if the electron density is less than a critical threshold^3^. Similarly, the maximization of distance under some constraints, such as in the Thomson problem of the distribution of electrons on the surface of a sphere, can lead to crystallization with topological defects^4^.

The problem of controlling and optimizing distance becomes much more complex and interesting in motile active and living systems^5^. A simple—one-dimensional—example is traffic flow on a highway. Here, distances between cars have to exceed the minimal breaking distance, which grows with increasing speed $v_0$, quadratically for the stopping distance, linearly in flowing traffic^6^. This implies an optimal distance to maximize flow, the product of speed and density^7^. In many living systems, where motion typically occurs in two or three spatial dimensions, distances between individuals should not be too large to facilitate mating and reproduction, and to collectively protect a group against predators^8–10^. At the same time, distances should not be too small so as not to hinder the search for food, or the individual motion, or even damaging collisions. Also, to prevent the spread of airborne infectious diseases, like COVID-19, it is important to maximize the distance to other individuals and to avoid crowded spaces^11–13^. However, recent studies of a model of active motion of finite-size particles with constant speed $v_0$ and slow rotational diffusion—called active Brownian particles (ABPs)—shows that activity can have the the opposite effect of motility-induced clustering and phase separation^14^. The origin of this behavior is the formation of small clusters by head-on collisions of a few particles, which only slowly disintegrate and thereby form the nucleus of larger clusters.

The essential difference between ABPs and living individuals, such as birds or pedestrians, is, of course, that the former are “dumb”, while the latter have a visual perception of their environment, and use this information to react by adapting their speed and direction of motion to avoid collisions. A pivotal issue revolves around the efficacy of individual pedestrians in upholding interpersonal distancing within dense crowds^15–17^. Recent controlled laboratory experiments with pedestrians moving in a room^18,19^ have cast light on the implications of factors such as pedestrian density, walking speed, and prescribed safety distances on interpersonal spacing within moderately crowded environments.

In important aspect in the emergence of collective behavior of intelligent agents with directional perception is the type of neighborhood of interaction, which is considered for decision making. Metric, topological, and “visual” neighborhoods have been considered. For bird flocks, there is clear evidence for topological interactions, where the nearest neighbors are considered independent of their distance, resulting in scale-free behavior of the flock^20^. It has also been argued that pure vision, *i.e.* the blocking of view on distant neighbors by close-by agents, plays a relevant role in swarm formation^21,22^. In the case of pedestrian, experiments indicate that a topological neighborhood can be ruled out, while results can be approximated by a metric neighborhood, but are best explained by a visual neighborhood that has elements of both, visual occlusion and metric distance^23^.

In this study, we aim to elucidate the physical mechanisms underlying the cognitive self-steering of pedestrians (or birds) in moderately dense crowds with nearly homogeneous spatial distribution. We consider a highly simplified model of cognitive self-steering particles (intelligent active Brownian particles, iABPs), which move with constant speed $v_0$, can sense their environment by visual perception, and react by applying a limit steering torque (“maneuverability”), but have no memory (and thus cannot estimate the speed of neighboring particles nor their direction of motion)^24–26^. Thus, our iABPs have to base their decisions on the redirection of motion entirely on the instantaneous position of their neighbors. Similar self-steering mechanisms have been considered in models of social interactions in animal groups^27^. We want to emphasize that our torque-based steering mechanism is different from the short-distance repulsion of some other swarming and flocking models, which employ a conservative repulsive interaction potential^28^. We perform extensive agent-based simulations to analyze iABP dynamics at finite density, in order to explore the complex interplay between particle density, activity level, maneuverability, vision angle, and vision range. Key factors such as the distance to the nearest neighbors, exposure time, and persistence in velocity direction are analyzed. The simulation results are compared with the results of recent experiments on pedestrians in a room, to gain insights to which extent our simple model is able to reproduce and explain pedestrian behavior under the imperative of maximizing distance.

## Results

### Model and simulation approach

We consider a system of *N* agents which are modeled as point particles. The equation of motion of particle *i* with position ${\varvec{r}}_{i}$ is

1 $$\begin{aligned} m \ddot{\textbf{r}}_i =- \gamma \dot{\textbf{r}}_i + {F}_{\text {act}} \textbf{e}_i. \end{aligned}$$

Here, *m* is the mass of the particle, $\gamma$ the translational friction coefficient, and ${F}_{\text {act}}$ the propulsion force along the instantaneous particle orientation $\textbf{e}_i$, resulting in the overdamped limit in a constant velocity $v_0=|F_{act}|/\gamma$. The self-steering behavior of each agent is affected by the positions of neighboring particles. Particle *i* can adjust its propulsion direction $\textbf{e}_i$ through self-steering in the direction $\textbf{u}_{ij} = (\textbf{r}_j - \textbf{r}_i)/|\textbf{r}_j - \textbf{r}_i|$, determined by its neighbors, with an adaptive torque $\textbf{M}^{av}_i$, as^24,26,29^

2 $$\begin{aligned} \dot{\textbf{e}}_i(t) = \textbf{M}^{av}_i + \varvec{\Lambda }_i(t) \times \textbf{e}_i(t), \end{aligned}$$

where $\varvec{\Lambda }_i$ represents Gaussian and Markovian stochastic processes with zero mean and correlations $\langle \varvec{\varLambda }_i(t) \cdot \varvec{\varLambda }_j (t') \rangle = 2(d-1)D_R \delta _{ij} \delta (t-t')$ in *d* spatial dimensions with rotational diffusion coefficient $D_R$.

The cognitive torque (referred to as the “visual” torque) acting on particle *i* is

3 $$\begin{aligned} \textbf{M}^{av}_i = -\frac{C_0}{N_{c,i}} \sum _{j\in VC} e^{-r_{ij}/R_0} \textbf{e}_i \times (\textbf{u}_{ij} \times \textbf{e}_i), \end{aligned}$$

where $C_0$ represents the “visual” maneuverability, and $N_{c,i}$ is the number of particles within the vision cone (VC). Particles within the VC are determined based on the condition $\textbf{u}_{ij} \cdot \textbf{e}_i \ge \cos (\theta )$, where $\theta$ is the vision angle, defining the opening angle of the vision cone centered on the particles’s orientation vector $\textbf{e}_i$. In addition, we limit the vision to $|{\varvec{r}}_i - {\varvec{r}}_j| \le R_V$, where $R_V > R_0$ is the vision range and treat all further apart particles as invisible, which is helpful for computational efficiency. Finally, the number of effectively visible particles in Eq. (3) is

4 $$\begin{aligned} N_{c,i} = \sum _{j\in VC}e^{-r_{ij}/R_0} . \end{aligned}$$

**Figure 1:**
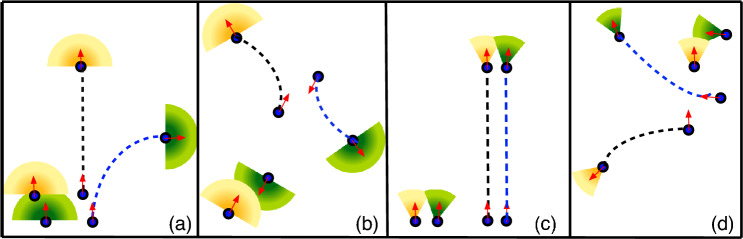
Schematic representation of two-particles interaction through visual perception and self-steering to avoid a close approach, as described by Eq. (3). The field of vision of the two particles is colored green and yellow, respectively, and corresponding trajectories are indicated by dashed lines. The initial configuration of the vision cones are illustrated near the starting points (with the initial propulsion direction indicated by opaque arrows) of the trajectories, while the final configurations accompany the trajectory lines. The effect of orientational noise on the trajectroies has been omitted for simplicity.

Here, some comments are in order. First, our choice of the steering torque in Eq. (3) is motivated by: (i) steering occurs in the plane defined by the unit distance vector $\textbf{u}_{ij}$ and the unit orientation vector $\textbf{e}_i$, (ii) the steering torque is a anti-symmetric function of the bearing angle $\Delta \phi$ between these two two vectors (i.e. target on the left, motion to the right, and vice versa), and (iii) it has an simple analytical form. This implies that the torque is a linear function of $\Delta \phi$ for small bearing angle, and thus vanishes for $\Delta \phi =0$; the only alternative would be a torque which has at least a discontinuity, or maybe even a singularity at $\Delta \phi =0$. Second, it is important to note that the steering torque, Eq. (3), is *non-additive*, due to the normalization by the visible particle number $N_{c,i}$. Third, the exponential range $R_0$ can be understood as a reduced effective vision range at higher local density of neighboring particles, which can arise from the partial occlusion of more distant neighbors by those close by^23,30^. The employ here the choice $R_V=4R_0$ if not stated otherwise. Finally, the steering torque in Eq. (3) implies an effective repulsive interaction, as illustrated schematically in Fig. 1.

In polar coordinates in two spatial dimensions, $\textbf{e}_i = (\cos \varphi _i , \sin \varphi _i)^T$, $\textbf{u}_{ij}= (\cos {\phi _{ij}}, \sin {\phi _{ij}})^T$ the equations of motion for the orientation angles $\varphi _i$ become

5 $$\begin{aligned} \dot{\varphi }_i = -\frac{C_0}{N_{c,i}} \sum _{j\in VC} e^{-r_{ij}/R_0} \sin ({\phi _{ij}-\varphi _i}) + \Lambda _i(t), \end{aligned}$$

where $\phi _{ij}$ is the angle under which particle *j* is seen by particle *i*, with bearing angle $\Delta \phi = \phi _{ij}-\varphi _{i}$. The sum on the right-hand side of Eq. (5) describes the tendency of a particles to move away from regions of high local particle density within its vision cone (VC).

In simulation, we measure time in units of $\tau _R$, length in units of $R_0$, but keep these units explicit in all expressions. The activity and steering of the agents is characterized by the Péclet number

6 $$\begin{aligned} \hbox{Pe} = \frac{v_{0}}{R_{0} D_{R}}, \end{aligned}$$

and the scaled maneuverability

7 $$\begin{aligned} \Omega = C_0/D_{R}, \end{aligned}$$

respectively. Both dimensionless numbers are ratios of the typical time scales of active and noisy motion. In the case of the Péclet number, these are the time scale $R_0/v_0$ of traversing the vision range $R_0$ with velocity $v_0$, and the rotational diffusion time $1/D_R$. Periodic boundary conditions of a square simulation box of linear extension *L* are employed to control the dimensionless particle density $\Phi =N (R_0/L)^2$. Compared to a system with explicit walls—unavoidable in experiments, like those with pedestrians^18^—this has the advantages that the system is completely homogeneous, and that particle motion over long distances can be analyzed.

We want to emphasize that (i) all particles move with constant velocity, no speed adaptation is considered, and (ii) no volume exclusion of particles is taken into account, in order to avoid jamming, which corresponds to systems for which the vision range is much larger than the particle size. Thus, our model applies to semi-dense crowds.

The simulations are performed in the over-damped limit, i.e. $m D_{R} /\gamma \ll 1$ , so that inertial effects are negligible. Explicitly, we choose $\gamma = 10^{2} D_{R}$ and $m=1$. The linear dimension of the simulation box is $L/R_{0} = 20$. We study systems with particle numbers $N=25$, 64, 100, and 225, which corresponds to densities $\Phi =0.0625$, 0.16, 0.25, and 0.5625, respectively. The equations of motion (1) are solved with a velocity-Verlet-type algorithm suitable for stochastic systems^31^, with the time step $\Delta t = 10^{-3} / D_{R}$.

### Distance to nearest neighbors

We analyze the probability distribution functions (PDFs) for the distance $d_1$ to the nearest neighbor, and extract information on the average $\langle d_1 \rangle$ and the fraction of particles closer than $R_0$ to other particles. We focus on the dependence on key parameters, like particle density $\Phi$, Péclet number $\hbox{Pe}=v_0/(R_0 D_R)$, maneuverability $\Omega$, and vision angle $\theta$.

#### Effect of particle density, activity, and maneuverability

**Figure 2:**
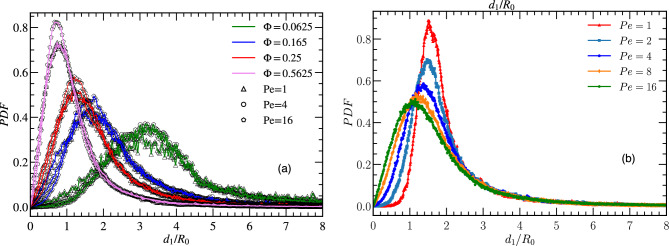
Probability density function (PDF) of the distance $d_1$ to the nearest neighbor. (**a**) For various particle densities $\Phi = 0.0625, 0.165, 0.25, 0.5625$ and activities $\hbox{Pe} \in {(1,4,16)}$, with fixed activity-to-maneuverability ratio $\hbox{Pe}^{3/2}/\Omega =1$, and vision angle $\theta =\pi /2$. (**b**) Dependence on Péclet number $\hbox{Pe}$, with fixed particle density $\Phi =0.25$, maneuverability $\Omega =16$, vision angle $\theta =\pi /2$, cutoff range $R_V= 4 R_{0}$.

Figure 2a shows the probability density functions (PDFs) of nearest-neighbor distance $d_1$ for various particle densities and activities $\hbox{Pe}$, for fixed vision angle $\theta =\pi /2$. The increase in the particle density from $\Phi =0.0625$ to $\Phi =0.5625$ results in a shift of the distribution towards lower distances $d_1$, indicating closer approaches between particles in more crowded environments. This results from a reduction in the inter-particle distance with density as $\Phi ^{-1/2}$—independent of particle mobility. Another interesting result is that a constant ratio $\hbox{Pe}^{3/2}/\Omega$ (in this case $\hbox{Pe}^{3/2}/\Omega =1$) results in a collapse of the distributions onto a single master curve, which indicates a tight coupling of individual activity and maneuverability. This is due to the requirement of higher steering torques for larger particle speed; a similar behavior has been found previously for pursuit dynamics, where scaling with $\hbox{Pe}/\Omega ^{1/2}$ is observed^25,32^. The $\hbox{Pe}^{3/2}/\Omega$ scaling will be discussed in more detail in the context of the average minimal distance $\langle d_1 \rangle$ below.

To study the effect of activity $\hbox{Pe}$, we analyze distance distribution $P(d_{1})$ at fixed $\Omega$ and particle density, see Fig. 2b for vision angle $\theta =\pi /2$, at various activities. Particles come closer to each other for larger $\hbox{Pe}$. Thus, slower-moving particles can maintain a larger distance because they can steer away from other particles already at a larger distance—at constant maneuverability.

Similar behavior is reported for pedestrians, where slower-moving can maintain a higher distance amongst themselves in comparison with faster-moving pedestrians^18^.

**Figure 3:**
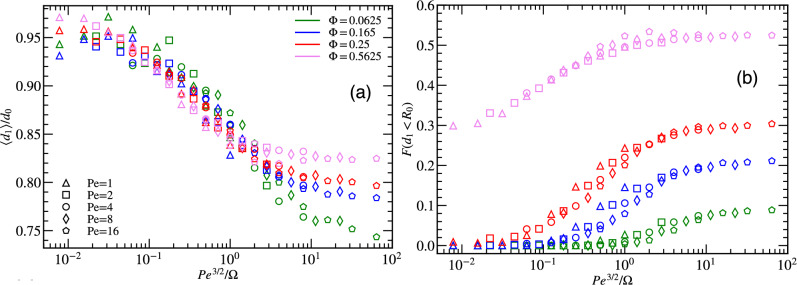
(**a**) Average nearest-neighbor distance $d_1$ for various pedestrian densities $\Phi$ as a function of $\hbox{Pe}^{3/2}/\Omega$. (**b**) Fraction of particles within a distance $R_{0}$ from other particles as a function of $\hbox{Pe}^\beta /\Omega$, with $\beta =3/2$, for various particle number *N*, as indicated. The vision angle in both cases is $\theta =\pi /2$.

From the PDF $P(d_{1})$, we can calculate the average minimal distance $\langle d_{1} \rangle$ to nearest neighbors, and the fraction of particles, $F(d_{1}<R_{0})$, which are at a distance to their nearest neighbors less than $R_{0}$. Figure 3a shows $\langle d_1 \rangle$, scaled with the neighbor distance $d_{0}= 2L/ \sqrt{\pi N'}$ in a regular triangular lattice with the same particle density. Here, we employ the effective particle number $N'=N+N_0$, with $N_0=3$ to account for finite-size effects and to improve the scaling. Figure 3a demonstrates that the data for $\langle d_1 \rangle /d_{0}$ as a function $\hbox{Pe}^{3/2}/\Omega$ collapse reasonably well onto a universal scaling curve, as expected from the scaling of $P(d_{1})$. Thus, the minimum distance $d_1$ decreases as the particle density increases as $\langle d_{1} \rangle \sim 1/\sqrt{N} \sim \sqrt{\Phi }$.

Furthermore, the results of Fig. 3a indicate that three dynamic regimes can be distinguished:

- The “overcautious distancing” regime [see movie M1^33^], for $\hbox{Pe}^{3/2}/\Omega \lesssim 0.05$, where particles keep a nearly constant distance from all neighbors, at the cost of hardly any translational motion,
- the “wiggling and squirming” regime [see movie M2], for $0.05 \lesssim \hbox{Pe}^{3/2}/\Omega \lesssim 10$, where steering helps particles to avoid each other while allowing significant persistent motion, and
- the “reckless motion” regime, for $\hbox{Pe}^{3/2}/\Omega \gtrsim 10$, where particles move without taking much—or any—notice of their neighbors.

In the “wiggling and squirming” regime, particles tend to approach each other closely before initiating steering maneuvers to avoid collisions, consequently leading to a reduction in the distance between the closest neighbors with increasing $\hbox{Pe}^{3/2}/\Omega$. The plateau of $\langle d_{1} \rangle /d_0$ observed in the “reckless motion” regime at $\hbox{Pe}^{3/2}/\Omega \gtrsim 10$, aligns with the measured values obtained from the ’non-interacting’ ABP simulations. This shows that for low maneuverability or high activity, agents do not react to each other and have limited scope to modify their movement direction.

Figure 3b displays the fraction *F* of particles, which have a distance less than $R_{0}$ to other particles. This fraction is examined for various particle densities and Péclet numbers $\hbox{Pe}$ for fixed maneuverability $\Omega$. The data for different $\hbox{Pe}$ also collapse onto a single master curve when plotted as a function of the scaling variable $\hbox{Pe}^{3/2}/\Omega$. The fraction *F* of close neighbors attains its maximum/minimum when the particle density is high/low—as to be expected because all distances decrease with increasing particle density. Furthermore, *F* is a monotonically increasing function of $\hbox{Pe}^{3/2}/\Omega$, consistent with behavior of $P(d_1)$ and $\langle d_{1} \rangle$. For large $\hbox{Pe}^{3/2}/\Omega$, the fraction *F* gradually approaches a plateau. The plateau values are approximately $F \simeq 0.1$ for $\Phi =0.0625$, 0.2 for $\Phi =0.165$, 0.3 for $\Phi =0.25$, and 0.55 for $\Phi =0.5625$. This saturation behavior can be attributed to a balance between the density of pedestrians, their movement characteristics, and the chosen threshold distance $R_{0}$. Once the fraction of close encounters near these limits, the additional increase in $\hbox{Pe}^{3/2}/\Omega$ has a diminishing effect on the fraction *F*.

#### Effect of vision angle

**Figure 4:**
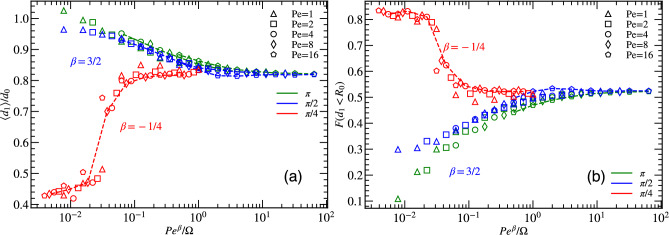
(**a**) Scaled average minimal distance $\langle d_1 \rangle$ for different vision angles $\theta$ as function of $\hbox{Pe}^{\beta }/\Omega$, where $\beta = 3/2$ for $\pi$ and $\pi /2$, and $\beta =-1/4$ for $\pi /4$. (**b**) Fraction *F* of particles within a distance $R_{0}$ from other particles for various vision angle $\theta$ and the $\hbox{Pe}^{\beta }/\Omega$ ratio. All data are for systems with particle density $\Phi =0.25$.

An important parameter of our model is the vision angle $\theta$. Results for the average minimal distance $\langle d_1 \rangle$ between particles are displayed in Fig. 4a as a function of $\hbox{Pe}^{\beta }/\Omega$, where the exponent $\beta$ is determined such as to optimize scaling with a single master curve. This yields $\beta \approx 3/2$ for vision angles $\pi$ and $\pi /2$, and $\beta \approx -1/4$ for vision angle $\pi /4$, for fixed particle density $\Phi =0.25$ and vision range $R_{V}=4R_{0}$. The behavior for large vision angle $\theta \ge \pi /2$ is found to be very different than for smaller vision angle $\theta =\pi /4$. For vision angle $\theta =\pi$, the behavior is essentially the same as for $\theta =\pi /2$ discussed above, see Fig. 3. In particular, there is a good data collapse with scaling variable $\hbox{Pe}^{3/2}/ \Omega$.

However, the situation changes quite dramatically for smaller vision angle $\theta =\pi /4$. Here, the restricted field of view limits the particle’s ability to detect each other from all directions. The restricted field of vision now does not always prevent collision, as particles can move toward each other with neither of them being able to the see the other. This can lead to very small particle separation. The minimal distance $\langle d_{1} \rangle / d_{0}$ now shows good data collapse with scaling variable $\hbox{Pe}^{-1/4}/\Omega$, see Fig. 4a. Note that since both $\hbox{Pe}$ and $\Omega$ contain a factor $1/D_R$, $\hbox{Pe}^{-1/4}/\Omega \sim D_R^{5/4}$, so that the scaling variable depends strongly on the rotational diffusion. This scaling with $\hbox{Pe}^{-1/4}/\Omega$ also implies that similar behavior is seen when $\hbox{Pe}$ and $\Omega$ are *inversely* proportional to each other, i.e. $\hbox{Pe}$ high, $\Omega$ low, and vice versa. The average minimal distance $\langle d_{1} \rangle /d_0$ attains a minimum for low $\hbox{Pe}^{-1/4}/\Omega$, which corresponds to high values of both maneuverability and activity (and small $D_R$). This behavior arises from collective motion, where particles are moving in parallel as a band, somewhat reminiscent of the motion of particles with alignment interactions in the Vicsek model. The reason is that for particles to stay in the band, they need a high persistence of motion to retain their parallel motion, and a high maneuverability to be able to quickly correct their direction of motion should their orientation deviate too much from parallelity. The particles in band-like structures for vision angle $\pi /4$ are in closer proximity to each other, which is also seen in the close-neighbor fraction *F*, see Fig. 4b. The fraction *F* decreases with decreasing $\Omega$ and $\hbox{Pe}$ (and increasing $D_R$). The band-like structures are characterized in more detail in Section “Band-like structure at narrow vision angles” below.

#### Effect of vision range

**Figure 5:**
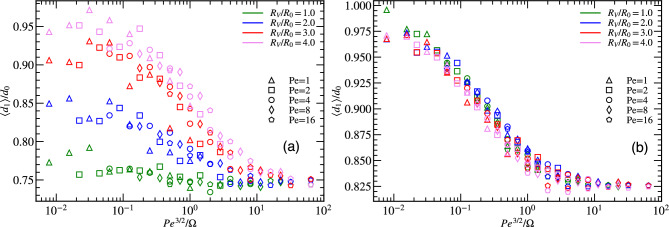
Average minimal distance $\langle d_1 \rangle$ for different $R_{V}$ and $\hbox{Pe}^{3/2}/\Omega$ for particle density (**a**) $\Phi =0.0625$ and (**b**) $\Phi =0.5625$. Vision angle $\theta =\pi /2$ for all cases.

In a model of visual-perception-induced steering, the vision range plays an essential role. Note that some effect of the vision range has already been absorbed into the definition of the dimensionless particle density $\Phi =N (R_0/L)^2$, where $R_0$ is the vision range at high local particle density. Thus, to elucidate the effect of vision range, we now vary the ratio $R_V/R_0$. Figure 5 shows the average minimal distance $\langle d_1 \rangle$ for various $R_V/R_0$ ratios at low and high particle densities at fixed vision angle $\pi$. The qualitative behavior is similar for different vision ranges $R_V$. For low density $\Phi =0.0625$, see Fig. 5a, $\langle d_1 \rangle$ for $\hbox{Pe}^{3/2}/\Omega \lesssim 1$ increases with increasing $R_V/R_0$, but saturates around $R_V/R_0 \simeq 4$. This happens because the effective density is now determined by $\Phi _V=N (R_V/L)^2$, with $\Phi _V = 16 \Phi$ for $R_V/R_0=4$. Thus, the system is effectively at much higher density for large $R_V$.

Conversely, in a dense system, see Fig. 5b, the effective vision range is essentially represented by $R_0$, because the exponential factor in Eq. (3) dominates, where particles beyond the distance $R_0$ hardly contribute. This leads a very weak dependence on the vision range $R_V$ already for $R_V/R_0 \gtrsim 1$.

### Exposure time

Another interesting quantity to consider is the exposure time $t_{m}$ i.e. the time spent by particles close to each other uninterruptedly. For our simulation, we chose again the distance $R_0$ to define proximity and scaled the exposure time with $D_{R}$. The scaled exposure time $T_{m}$ is given as :

8 $$\begin{aligned} T_{m}= t_m/D_{R}. \end{aligned}$$

**Figure 6:**
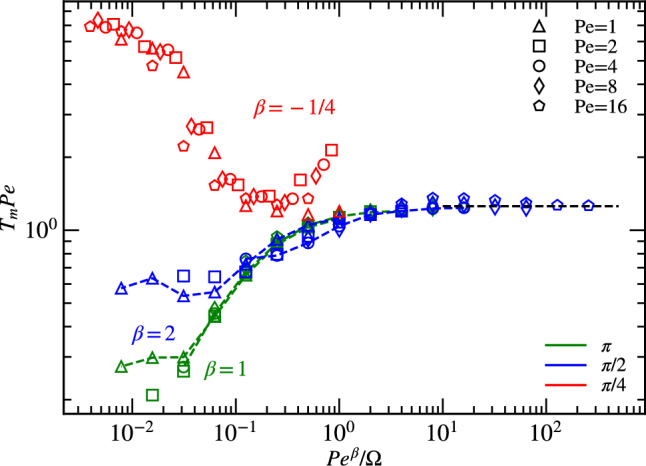
Scaled average exposure time, $T_m \hbox{Pe}$, during which particles remain close to each other within a vicinity of radius $R_0$ uninterruptedly, for various Péclet numbers (Pe), as indicated $\Phi =0.25$.

Figure 6 displays the dependence of the scaled average exposure time $T_{m} \hbox{Pe}$ on the dimensionless ratio $\hbox{Pe}^{\beta }/\Omega$ for particle density $\Phi =0.25$. The relationship is studied for vision angles $\theta =\pi$, $\pi /2$, and $\pi /4$. The value of the exponent $\beta$, determined by good data collapse for different $\hbox{Pe}$ and $\Omega$, depends on the vision angle, with $\beta =1$ for $\theta =\pi$, $\beta =2$ for $\theta =\pi /2$, and $\beta =-1/4$ for $\theta =\pi /4$. The qualitatively different scaling for $\theta \ge \pi /2$ and $\theta =\pi /4$ has the same origin as the scaling of the average minimal distance $\langle d_1 \rangle$ in Fig. 4.

Notably, the scaled exposure time becomes nearly independent of particle density or vision angle for $\hbox{Pe}^{\beta }/\Omega \gtrsim 1$, with

9 $$\begin{aligned} T_{m} \hbox{Pe} = A. \end{aligned}$$

The constant *A* can be calculated in the “ideal gas” limit of nearly straight particle trajectories, by considering the length of segments of straight lines intersecting a circle, with a homogeneous distribution of perpendicular distances from the circle center. This yields $A=A_{id}=\pi /2$, in reasonable agreement with the data in Fig. 6 for large $\hbox{Pe}^{\beta }/\Omega$. This indicates for $\theta =\pi$ and $\pi /2$ that the exposure time is nearly independent of steering and maneuverability at high particle velocities, and inversely proportional particle velocities $\hbox{Pe}$.

Therefore, the interesting behavior, where particles can react to their environment by steering their motion, occurs for $\hbox{Pe}^{\beta }/\Omega \lesssim 1$. For larger vision angle $\theta =\pi$ and $\pi /2$, the results in Fig. 6 indicate that at higher maneuverability and lower activity, particles can steer well away from each other, so that the exposure time is very low. The exposure time is smaller for $\theta =\pi$ compared to $\theta =\pi /2$, which indicates better steering for particle avoidance. For the smaller vision angle $\theta =\pi /4$, the functional dependence of the exposure time reflects again the qualitatively different behavior discussed above, with a maximal exposure time for $\hbox{Pe}^{-1/4}/\Omega \lesssim 3\times 10^{-2}$.

The dependence of the average exposure time on activity, maneuverability, vision angle, and particle density, reflects the motion and steering mechanisms discussed in the previous subsections. In particular, the prolonged exposure time for $\theta =\pi /4$ low $\hbox{Pe}^{-1/4}/\Omega$ can be attributed to collective motion in the form of bands within this regime. (for details see Section “Band-like structure at narrow vision angles”). Density has only a weak effect on exposure time, with somewhat longer exposure time at higher densities. This is due to the definition of exposure time, where only particle pairs contribute which are within the $R_0$ vision range.

### Mean-square displacement

The translational motion of the active Brownian particles is characterized by their mean-square displacement (MSD)

10 $$\begin{aligned} \langle {\varvec{r}}^{2}(t) \rangle = \frac{1}{N} \sum _{i=1}^N \left\langle \left( {\varvec{r}}_i(t+t_0)- {\varvec{r}}_i(t_0) \right) ^2 \right\rangle , \end{aligned}$$

where the average is performed over the initial time $t_0$. The theoretical calculations in two dimensions for active Brownian particles yield^5,34,35^

11 $$\begin{aligned} \langle {\varvec{r}}^{2}(t) \rangle =4D_T t + \frac{2v_0^2}{D_R^2} \left( D_R t -1 + e^{-D_R t}\right) . \end{aligned}$$

**Figure 7:**
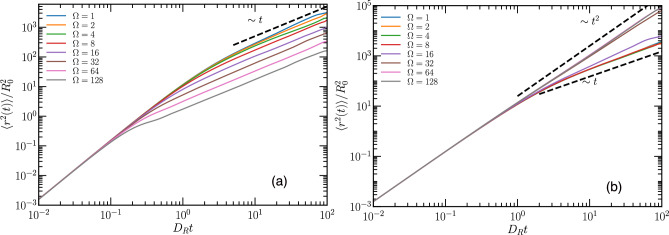
Mean squared displacement of particles at $\Phi =0.25$ and $\hbox{Pe}=4$ for various maneuverabilities $\Omega$, as indicated, for vision angle (**a**) $\theta =\pi$ and (**b**) $\pi /4$.

Figure 7a displays the time dependence of the mean-square displacement for various maneuverabilies, with vision angle $\theta =\pi$ and fixed activity $\hbox{Pe}=4$. The particles exhibit short-time ballistic and long-time diffusive behavior, where the effective translational diffusion coefficient decreases with increasing maneuverability. The particles behave very similarly to free Active Brownian Particles (ABPs) for small maneuverability $\Omega =1$, while their diffusion is strongly reduced for large maneuverability $\Omega =128$, where particles are overly cautious in their movement and try to avoid the vicinity of their neighbors.

For vision angle $\pi /4$, the MSD displays two different power laws for long times, depending on the maneuverability, see Fig. 7b. For low maneuverability $\Omega \le 8$, the MSD curves overlap, and the long-time MSD is diffusive with $\hbox{MSD} \sim t$. This behavior is typical of free ABPs. However, at higher maneuverability $\Omega \ge 32$, the long-time MSD is ballistic, with $\hbox{MSD} \sim t^2$. The latter case corresponds to the regime of $\hbox{Pe}^{-1/4}/\Omega \lesssim 3\times 10^{-2}$ in Figs. 4 and 6, where band formation and collective motion of particles emerges.

**Figure 8:**
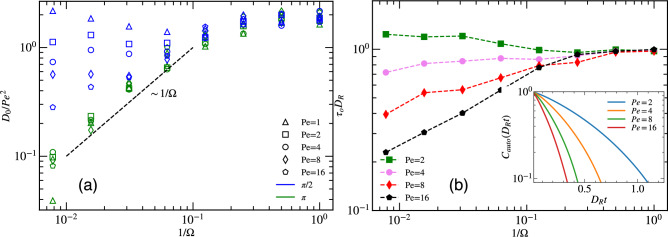
(**a**) Scaled effective long-time diffusion constant $D_{0}=D_{eff}/D_{R}$ extracted from the MSD at various $\hbox{Pe}$, as indicated, for article density $\Phi =0.25$. (**b**) Relaxation time $\tau _{0}$ of particle motion direction as function of maneuverability $\Omega$, at vision angle $\theta =\pi /2$ and particle density $\Phi =0.25$, for various $\hbox{Pe}$. Inset: Auto-correlation function of the propulsion direction of individual particles at $\Omega =64$, vision angle $\theta =\pi /2$, and particle density $\Phi =0.25$.

From the mean-square displacement (MSD) curve, we can derive the effective long-time diffusion constant for both vision angles $\pi$ and $\pi /2$, where we observe diffusive behavior. Results are presented in Fig. 8a as a function of maneuverability for particle density, $\Phi =0.25$, and various activities. To set the value of the diffusion coefficient $D_0= D_{eff}/D_{R}$ into perspective, we scale it with $\hbox{Pe}^2$, which corresponds to the behavior of free ABPs, compare Eq. (11). Remarkably, data for different $\hbox{Pe}$ then collapse onto each other for small maneuverability $1/\Omega \ge 0.3$. The effect of particle density was not significant and qualitative similar behavior is obtained at higher density (see SI Fig. S2).

For systems with vision angle $\theta =\pi$, we observe a remarkable convergence of data points for different $\hbox{Pe}$. As $1/\Omega$ decreases, the effective diffusion coefficient scales as $D_{0}/\hbox{Pe}^2 \sim 1/\Omega$, resulting in the universal behavior $D_{0} \sim \hbox{Pe}^{2}/\Omega$, i.e., $D_{eff} \sim v_{0}^2/C_{0}$ independent of rotational diffusion. In contrast, data points for vision angle $\theta =\pi /2$ are very scattered for $1/\Omega \lesssim 0.3$, and no scaling behavior emerges.

### Temporal auto-correlation function

The temporal auto-correlation function of individual particles is given by

12 $$\begin{aligned} C_{auto}(t) = \frac{1}{N} \sum _{i=1}^N \left\langle {\varvec{e}}_i(t+t_0) \cdot {{\varvec{e}}}_i(t_0) \right\rangle , \end{aligned}$$

where ${\varvec{e}}_i$ is the orientation of particle *i*, and *N* is the total number of particles. The inset of Fig. 8b shows the auto-correlation function of particles in the strong-steering regime. The decay becomes faster with increasing Péclet number. Interestingly, this is in contrast to simple ABPs, where the relaxation time is independent of $\hbox{Pe}$. This $\hbox{Pe}$-dependence is due to the closer encounters of particles at higher activity, which imply rapid changes of their orientation. Similar results were reported in the pedestrians experiment of Ref.^18^, where the direction of motion of faster-moving pedestrians also relax faster.

The relaxation time $\tau _{0}$ can be extracted from the initial exponential decay of the auto-correlation function,

13 $$\begin{aligned} \sum _{i=1}^N \left\langle {{\varvec{e}}}_i(t+t_0) \cdot {{\varvec{e}}}_i(t_0) \right\rangle = A \exp ({-t/\tau _{0}}). \end{aligned}$$

Figure 8b illustrates the dependence of the relaxation time $\tau _{0}$ on $\hbox{Pe}$ and $\Omega$. For lower maneuverability, $1/\Omega \ge 0.1$, particles are in the ABP regime and hence the relaxation time is completely determined by rotational diffusion constant $D_{R}$. For higher maneuverabilities, where particles can steer effectively away from each other, the value of relaxation time is determined by both activity and maneuverability. Higher maneuverability results in stronger steering and consequently faster reorientation and relaxation and the propulsion direction.

### Local particle distributions and trajectories

**Figure 9:**
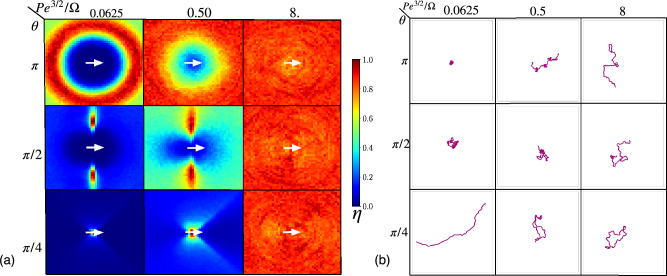
(**a**) Density distribution $\eta$ (normalized with the maximum value) of particles around a given particle whose orientation is as indicated by the small white arrow to the right at the center, for various vision angles $\theta$ and activity-maneuverability ratios $\hbox{Pe}^{3/2}/\Omega$, as indicated. (**b**) Exemplary trajectories paths of active self-steering particles are shown for different vision angles $\theta$ and Péclet-maneuverability ratios $\hbox{Pe}^{3/2}/\Omega$, as specified. All results are shown for $\hbox{Pe}=4$.

To further characterize typical particle conformations and dynamics, we consider the density distribution in a particle-centered and oriented reference frame for various ratios $\hbox{Pe}^{3/2}/\Omega$ and vision angles $\theta$, see Fig. 9a. For vision angle $\theta =\pi$, the particle distribution is isotropic; for small $\hbox{Pe}^{3/2}/\Omega =0.0625$, there is a pronounced density peak at the vision range $R_V$, indicative of high steering maneuverability, where particles are able to maintain a distinct separation from one another. This peak is smeared out and disappears with increasing $\hbox{Pe}^{3/2}/\Omega$. For vision angles $\theta =\pi /2$ and $\pi /4$, due to the asymmetry in the vision field, the density distribution also becomes highly asymmetric for small and moderate $\hbox{Pe}^{3/2}/\Omega$, with less number of particles in front and back, and more particles in the side-wise direction.

Figure 9b displays corresponding representative trajectories. For vision angle $\theta =\pi$ and high maneuverability, with $\hbox{Pe}^{3/2}/\Omega < 0.1$, particles remain almost stationary, just wiggling around their average location. As the vision angle decreases, and $\hbox{Pe}^{3/2}/\Omega$ increases, particle become more mobile, and trajectories more persistent. Notable is the motions for $\theta =\pi /4$, where particles exhibit nearly straight and extended trajectories, which arises from the pronounced directional motion due to the formation of band-like structures (see Section “Band-like structure at narrow vision angles”).

### Band-like structure at narrow vision angles

**Figure 10:**
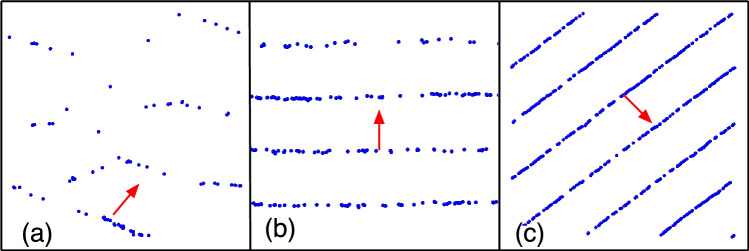
Snapshots showing band-like motion for vision angle $\pi /4$, for $\hbox{Pe}=4$, $\Omega =16$, $R_{V}/R_{0}=4.0$, and $\hbox{Pe}^{3/2}/\Omega =0.125$, for particle’s density (**a**) $\Phi =0.25$, (**b**) $\Phi =0.625$ and (**c**) $\Phi =2.5$. The red arrow indicates the propagation direction of the particle. See also Movies M3, M4.

As noted above, band-like aggregates and motion patterns appear for low activity-maneuverability ratio $\hbox{Pe}^{3/2}/\Omega =0.125$ and narrow vision angle $\theta = \pi /4$, somewhat reminiscent of the bands in the Vicsek model near the transition from the polarized to the disordered phase^36–38^. However, due to the different types of interactions, these bands are very thin, consisting only of a single line of particles, compared to the bands in the Vicsek model. The restricted vision implies that the particles can only react to and interact with other particles in front of them, but are not aware of or responsive to particles on their sides in perpendicular directions; thus, the particles can come very close to each other, with small distances to the nearest neighbors $\langle d_1 \rangle$ (see Section “Distance to nearest neighbors”) and a large exposure times $T_{m}$ (see Section “Exposure time”). Figure 10 shows typical snapshots of band-like structures at different particle densities. When the particle density is low, $\Phi = 0.25$, the band-like structures are not very prominent, because the particles have more available space to move around, allowing for more freedom of motion. However, as the density increases, the available space per particle decreases, and the band-like structures become much more distinct, even forming a one-dimensionally ordered stripe phase at $\Phi =2.5$.

The dependence of the stationary-state conformations of the bands on maneuverability and Péclet number is characterized below. We want to mention parenthetically that the coarsening dynamics of band formation is also very interesting, see SI, Section S-III and Movie M5. Starting from a random initial condition, we observe first the formation of small bands, with an isotropic distribution of the direction of motion. Therefore, these bands often collide, mostly pass through each other, but at the same time also slightly adjust their direction of motion. In this way, bands merge and grow, until they move all collectively in the same direction.

#### Polarization

**Figure 11:**
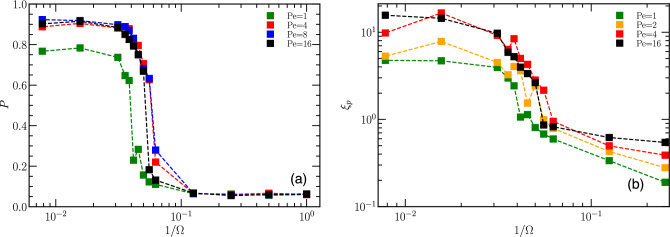
(**a**) Polarization *P* as a function of maneuverability $\Omega$ at vision angle $\pi /4$, density $\Phi =0.625$ at various $\hbox{Pe}$, as indicated. The sharp drop of *P* at $1/\Omega \simeq 0.05$ indicates a transition from the uniformly distributed, randomly moving ABPs at low $\Omega$ to collective motion in the form of bands at high $\Omega$. (**b**) Persistence length $\xi _{p}$ extracted from the spatial correlation function for $\Phi =0.625$, and vision angle $\pi /4$, for various indicated $\hbox{Pe}$ as a function of maneuverability $\Omega$, showing the transition from randomly moving ABPs having low persistence motion to highly persistence collective motion.

We characterize the transition from the state of disordered motion to band formation by the global polarization order parameter^29,39^

14 $$\begin{aligned} P= \left\langle \frac{1}{N} \left| \sum _i \textbf{e}_i \right| \right\rangle , \end{aligned}$$

where ${{\varvec{e}}}_i$ is orientation of particle *i* and the average is performed over time. Figure 11a illustrates the polarization *P* as a function of maneuverability $\Omega$, at particle density of $\Phi =0.625$. At low maneuverability, i.e. for $1/\Omega \ge 1/8$, particles display random orientations, resulting in polarization $P \approx 0$. However, as maneuverability increases, a transition occurs at $1/\Omega \simeq 1/16$, where particles align their orientations and a banded state with large global polarization emerges. As $\Omega$ increases further, the polarization nearly reaches unity, in particular for $1/\Omega \le 1/32$, and larger $\hbox{Pe}$.

#### Spatial correlations and persistence length

Another interesting quantity to characterize the banded state is the spatial correlation function^40,41^,

15 $$\begin{aligned} C_{e}({\varvec{r}}) =\left\langle \frac{\sum _{i,j\ne i}{\varvec{e}}_i \cdot {\varvec{e}}_j \delta (\varvec{r}-({\varvec{r}}_i-{\varvec{r}}_j))}{\sum _{i,j\ne i}\delta ({\varvec{r}}-({\varvec{r}}_i-{\varvec{r}}_j))}\right\rangle , \end{aligned}$$

where ${\varvec{e}}_i$ and ${\varvec{e}}_j$ represent the orientation vectors for particle *i* and particle *j*, respectively. The spatial correlation function can be used to extract the information about the persistence length $\xi _p$ as

16 $$\begin{aligned} \left\langle \sum _{i,j\ne i} {\varvec{e}}_i \cdot \varvec{e}_j \delta ({\varvec{r}}-({\varvec{r}}_i-{\varvec{r}}_j)) \right\rangle = B \exp ({-|{\varvec{r}}_{i}-{\varvec{r}}_j|/ \xi _p}). \end{aligned}$$

The persistence length of the band-like structures is shown in Fig. 11b for different activities $\hbox{Pe}$ and maneuverabilities $\Omega$, at $\Phi =0.625$. A transition from a high-persistence-length phase at $1/ \Omega \le 1/32$ to a low-persistence-length phase at $1/ \Omega \ge 1/16$ is evident, similar to the behavior observed for global polarization, see Section “Band-like structure at narrow vision angles”. The dependence on the Péclet number ($\hbox{Pe}$) appears to be relatively weak, with $\xi _p$ decreasing with decreasing $\hbox{Pe}$.

## Discussion

We have analyzed the behavior of active, self-steering particles with visual perception in semi-dilute crowds, where each particle self-steers to avoid regions of high neighbor density in their vision cone. We focus on a minimal model, where particles move with constant velocity and have only instantaneous spatial information of their neighbors.

The dependence of the probability density function (PDF) of the minimal distance $d_1$ to neighboring particles, the fraction of particles in the close vicinity of neighbors, as well as the expose time on particle Péclet number $\hbox{Pe}$, maneuverability $\Omega$, vision angle $\theta$, and density $\Phi$ is investigated. We find that the PDF $P(d_1)$ displays a peak, which shifts toward lower value with increasing particle density. Furthermore, PDFs for constant $\hbox{Pe}^{3/2}/\Omega$ ratio display universal scaling behavior, which indicates that stronger maneuverability is required at high activities to avoid close contact; similar features were observed in previous studies of iABP^25^, and iAOUP (intelligent Active Ornstein Uhlenbeck particles) pursuit dynamics^32^, where self-steering is *toward* regions of high local particle density in the vision cone.

We also examined the impact of the vision angle $\theta$, which reveals that particles with wider fields of view (larger $\theta$) are better equipped to detect potential collisions and steer away from potential collisions earlier, resulting in a larger minimum distance $d_1$. Conversely, particles with narrower fields of view have shorter minimum distances. For narrow vision cone (vision angle $\theta =\pi /4$), we observe the formation of band-like structures and collective motion for high maneuverability strength, somewhat reminiscent of the bands in the Vicsek model.

For the duration that particles spent in close proximity to other particles, known as exposure time $T_{m}$, we find a correlation between high levels of particle activity and short exposure time. For large vision angles $\pi$ and $\pi /2$, the scaled exposure $T_m \hbox{Pe}$ show a consistent universal behavior as function the activity-maneuverability ratio $\hbox{Pe}^{\beta }/\Omega$, with $\beta =1$ and $\beta =2$ respectively. For small vision angle $\pi /4$, exposure time is very high at high maneuverability and is characterized by a negative exponent $\beta =-1/4$, due to the formation of band-like structures. The motion of agents within these bands is highly persistent, as indicated by their trajectories and persistence lengths. Furthermore, these bands are associated with a highly polarized state, characterized by a polarization order parameter $P\approx 1$.

The results of our model system can be compared—to some extent—to those of recent experiments of walking pedestrians confined in a room, with the goal to maintain a large “safety” distance to other pedestrians^18^. Several of our results are in good qualitative agreement with the experimental observations. As the pedestrian density increases, interactions become more frequent, leading to smaller distances between them. Additionally, more briskly walking pedestrians exhibit reduced minimum distances, as higher activity requires pedestrians to approach others more closely before steering becomes effective to avoid collisions^18^. The experimental results on fast-moving pedestrians also reveal similar features of exposure time as in our simulations, with an exposure time that is inversely proportional to walking speed^18^. We also observe faster relaxation of orientation direction for higher particles activity, in good agreement with experiments on pedestrians^18^.

We want to emphasize that our model has of course several limitations in describing the behavior of real pedestrians. One limitation is the idealization of constant speed, while pedestrians can adapt their speed. Another is that we consider instantaneous spatial information only, while pedestrians are able to judge the motion direction and speed of their neighbors, extrapolate to future collision points, and adjust their motion accordingly^42,43^. However, such extrapolations become increasingly difficult as the number of particles in the vision cone, the extrapolation time, and thereby the number of potential near-collisions increase. As the number of number of potential paths to be considered grows very rapidly, it has been suggested that the information entropy of hypothetical trajectories, where particles gain knowledge about the location of other particles or confining boundaries, could be employed to choose a short-time motion which maximizes the diversity of possible future, longer-time trajectories^44^. Which of these model provides a good description of collective pedestrian motion remains to be elucidated.

Overall, our study sheds light on the complex interplay between particle behavior, activity levels, vision angles, and other parameters. For large vision angles, like $\theta =\pi$ and $\pi /2$, the results of our model can qualitatively match some behavior of pedestrian crowds like mean exposure time and the probability distribution function of distance to the nearest neighbor. It would certainly be interesting to study the distance distributions of in other animal swarms more quantitatively, where flocks of birds and swarms of insects look like promising candidates.

It would now of course be very interesting to apply our model to other situations of the dynamics of pedestrian crowds, such as crossing or evacuation scenarios, and to compare the results with corresponding pedestrian experiments^19,45,46^. This is an interesting topic for future studies with an extended version of our model.

### Supplementary Information

Supplementary Information 1.

Supplementary Information 2.

Supplementary Information 3.

Supplementary Information 4.

Supplementary Information 5.

Supplementary Information 6.

## Data Availability

The data that support the findings of this study are available from the corresponding author upon reasonable request.
