# Supplementary material for: Controlling inter-particle distances in crowds of motile, cognitive, active particles
Source: Sci Rep. 2024 Apr 24;14:9443. doi: 10.1038/s41598-024-59022-6 (PMC11043455; doi:10.1038/s41598-024-59022-6)
Supplement: Supplementary file 6 — Supplementary Information 6. [file 41598_2024_59022_MOESM6_ESM.pdf]

Controlling Inter-Particle Distances in Crowds of Motile, Cognitive, Active Particles

Rajendra Singh Negi,[1,*] Priyanka Iyer,[1,†] and Gerhard Gompper[1,‡]

[1] *Theoretical Physics of Living Matter, Institute of Biological Information Processing and Institute of Advanced Simulation, Forschungszentrum Jülich, 52425 Jülich, Germany*

S-I. EXPOSURE TIME

Figure S1 illustrates the dependence of the scaled average exposure time $T_m Pe$ on the dimensionless ratio Pe^{β}/Ω for particle density $\Phi = 0.5625$. The relationship is investigated for vision angles $\theta = \pi$, $\pi/2$, and $\pi/4$. The exponent β is determined by achieving a good data collapse for various Pe and Ω, which is dependent on the vision angle. Specifically, $\beta = 1$ when $\theta = \pi$, $\beta = 2$ when $\theta = \pi/2$, and $\beta = -1/4$ when $\theta = \pi/4$. Here again, the scaled exposure time becomes nearly independent of particle density or vision angle for $Pe^{\beta}/\Omega \gtrsim 1$, with $T_m Pe = A$. This behavior is similar at lower density (see main text Sec III-B).

S-II. EFFECTIVE DIFFUSION CONSTANT

We extracted the effective long-time diffusion constant at higher density $\Phi = 0.625$. Figure S2 show the effective diffusion D_0 as function of maneuverability Ω. We observed a similar trend as we get at lower density (see main text III-C). For systems with a vision angle of $\theta = \pi$, we observe a remarkable convergence of data points across various Pe values. As $1/\Omega$ decreases, the effective diffusion coefficient scales as $D_0/P_e^2 \sim 1/\Omega$, leading to a universal behavior where $D_0 \sim Pe^2/\Omega$, specifically, $D_{\text{eff}} \sim v_0^2/C_0$, independent of rotational diffusion. In contrast, data points for a vision angle of $\theta = \pi/2$ exhibit significant scattering for $1/\Omega \lesssim 0.3$, and no discernible scaling behavior emerges. For lower values of maneuverabilities, the values saturate to $2D_R$ which is the ABP limit.

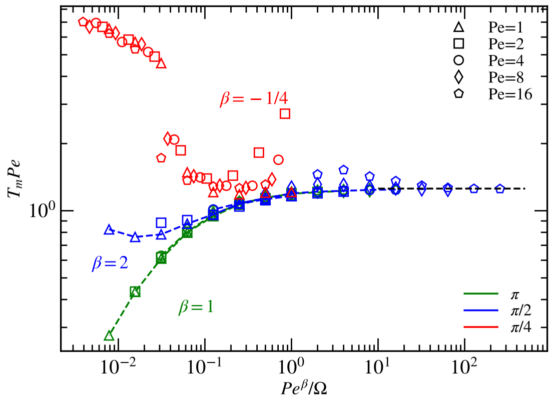

FIG. S1. Scaled average exposure time, $T_m Pe$, during which particles remain close to each other within a vicinity of radius R_0 uninterruptedly, for various Péclet numbers (Pe), as indicated. Particle density is $\Phi = 0.625$.

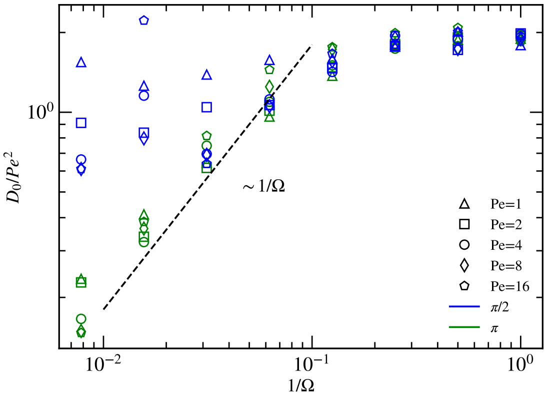

FIG. S2. Effective long-time diffusion constant extracted from the MSD at various indicated Pe, for particle density $\Phi = 0.625$.

S-III. DYNAMICS OF BAND-LIKE STRUCTURE FORMATION

An interesting issue is also the coarsening dynamics as the bands emerge from an initially disordered state. Figure S3 shows some snapshots of this temporal evolution of band-like structures for particle density $\Phi = 2.5$. All particles are initially randomly distributed in the entire simulation space (see Fig. S3(a)). As time progresses, small clusters and band-like structures form rather quickly (see Fig. S3(b)). These small bands still move in all directions and can pass through each other as they collide (see Fig. S3(c)). At the same time, bands reorient upon collisions and continue to grow, until the fully ordered state of parallel bands emerges (see Fig. S3(d)).

* r.negi@fz-juelich.de
† p.iyer@fz-juelich.de
‡ g.gompper@fz-juelich.de

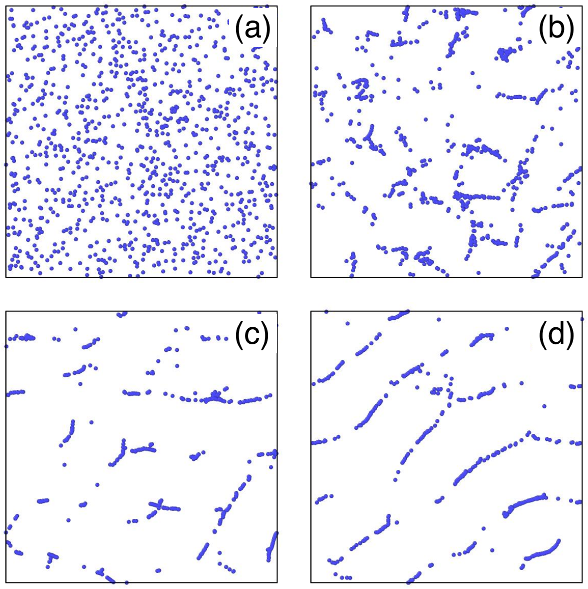

FIG. S3. The temporal progression of band-like structures from (a) an initial random configuration, to (b) small and (c) larger bands, crossing through each other and merging, to reach (d) the uni-directionally moving ordered bands. See also movie M5.

S-IV. MOVIE CAPTIONS

Movie M1: The dynamics of "overcautious distancing" regime particles at Péclet number $Pe = 4$, maneuverability $\Omega = 128$, particle density $\Phi = 0.25$, and vision angle π. The trajectory of one particle is shown.

Movie M2: The dynamics of particles in"wiggling and squirming" regime at Péclet number $Pe = 4$, maneuverability $\Omega = 16$, $\Phi = 0.25$, and vision angle $\pi/2$.

Movie M3: Band like motion of particles at particle density $\Phi = 0.5625$, Péclet number $Pe = 4$, maneuverability $\Omega = 128$, and vision angle $\pi/4$.

Movie M4: Band-like structures look like ordered strips of particles at particle density $\Phi = 2.5$, Péclet number $Pe = 4$, maneuverability $\Omega = 128$, and vision angle $\pi/4$.

Movie M5: Temporal evolution of band-like structures from a random initial configuration, for particle density $\Phi = 2.5$, Péclet number $Pe = 4$, maneuverability $\Omega = 128$, and vision angle $\pi/4$.
